# Dietary Patterns and Clinical Outcomes in Hemodialysis Patients in Japan: A Cohort Study

**DOI:** 10.1371/journal.pone.0116677

**Published:** 2015-01-21

**Authors:** Kazuhiko Tsuruya, Shingo Fukuma, Takafumi Wakita, Toshiharu Ninomiya, Masaharu Nagata, Hisako Yoshida, Satoru Fujimi, Yutaka Kiyohara, Takanari Kitazono, Kazuhiro Uchida, Tomoko Shirota, Tadao Akizawa, Takashi Akiba, Akira Saito, Shunichi Fukuhara

**Affiliations:** 1 Department of Integrated Therapy for Chronic Kidney Disease, Graduate School of Medical Sciences, Kyushu University, Fukuoka, Japan; 2 Department of Healthcare Epidemiology, Kyoto University Graduate School of Medicine and Public Health, Kyoto, Japan; 3 Institute for Health Outcomes and Process Evaluation Research (iHope International), Tokyo, Japan; 4 Faculty of Sociology, Kansai University, Osaka, Japan; 5 Department of Medicine and Clinical Science, Graduate School of Medical Sciences, Kyushu University, Fukuoka, Japan; 6 Community Medicine Education Unit, Graduate School of Medical Sciences, Kyushu University, Fukuoka, Japan; 7 Fukuoka Renal Clinic, Fukuoka, Japan; 8 Department of Environmental Medicine, Graduate School of Medical Sciences, Kyushu University, Fukuoka, Japan; 9 Department of Health Promotion, School of Health and Nutrition Sciences, Nakamura-Gakuen University, Fukuoka, Japan; 10 Division of Nephrology, Department of Medicine, Showa University School of Medicine, Tokyo, Japan; 11 Department of Blood Purification and Internal Medicine, Kidney Center, Tokyo Women’s Medical University, Tokyo, Japan; 12 Division of Nephrology and Dialysis Center, Shonantobu General Hospital, Kanagawa, Japan; 13 Center for Innovative Research for Communities and Clinical Excellence, Fukushima Medical University, Fukushima, Japan; University College London, UNITED KINGDOM

## Abstract

**Background & Objectives:**

Little is known about actual dietary patterns and their associations with clinical outcomes in hemodialysis patients. We identified dietary patterns in hemodialysis patients in Japan and examined associations between dietary patterns and clinical outcomes.

**Design, setting, participants, measurements:**

We used data from 3,080 general-population participants in the Hisayama study (year 2007), and data from 1,355 hemodialysis patients in the Japan Dialysis Outcomes and Practice Patterns Study (JDOPPS: years 2005–2007). Food intake was measured using a brief self-administered diet-history questionnaire (BDHQ). To identify food groups with the Hisayama population data, we used principal components analysis with Promax rotation. We adjusted the resulting food groups for total daily energy intake, and then we used those adjusted food-group scores to identify dietary patterns in the JDOPPS patients by cluster analysis (Ward’s method). We then used Cox regression to examine the association between dietary patterns and a composite of adverse clinical outcomes: hospitalization due to cardiovascular disease or death due to any cause.

**Results:**

We identified three food groups: meat, fish, and vegetables. Using those groups we then identified three dietary patterns: well-balanced, unbalanced, and other. After adjusting for potential confounders, we found an association between an unbalanced diet and important clinical events (hazard ratio 1.90, 95% C.I. 1.19–3.04).

**Conclusions:**

Hemodialysis patients whose diet was unbalanced were more likely to have adverse clinical outcomes. Thus hemodialysis patients might benefit not only from portion control, but also from a diet that is well-balanced diet with regard to the food groups identified here as meat, fish, and vegetables.

## Introduction

Dietary management is important to improve outcomes in hemodialysis patients. Clinical guidelines provide a recommended intake of micronutrients[1] to prevent hyperphosphatemia, hyperkalemia, hypertension, and water retention. Reduced intakes of protein, raw vegetables, and salt are recommended.[2–8] Excessive dietary restriction may of course result in malnutrition, but details of dietary patterns that might improve outcomes in hemodialysis patients are largely unknown.

Some previous research on nutritional epidemiology in kidney disease has focused on the absolute amounts of foods and micronutrients[7,9]. We focused instead on dietary patterns, which were identified by their balance (or unbalance) among food groups. Given that the prognosis of hemodialysis patients is better in Japan than in the US and Europe, we expected that an understanding of the relationship between dietary pattern and prognosis in hemodialysis patients in Japan would also provide useful information for hemodialysis care in other countries.

Here we report the results of a cohort study using data from hemodialysis patients participating in the Japan Dialysis Outcomes and Practice Patterns Study (JDOPPS) [10,11]. Our goals were to identify dietary patterns in those patients and to investigate relationships between dietary patterns and important clinical outcomes.

## Methods

### Ethics

The ethics committees of Kyushu University (Fukuoka, Japan) and Kyoto University (Kyoto, Japan) approved this study. Written informed consent was obtained from participants in the Hisayama study[12,13] and in the JDOPPS. The data were analyzed anonymously.

### Participants and setting

The participants were selected from among Japanese volunteers participating in the Hisayama study[12,13] and Japanese hemodialysis patients participating in the JDOPPS.

The Hisayama study is a population-based study that has been conducted since 1961 in Hisayama-cho in the Kyushu region of Japan. Subjects are volunteers of various ages, and are considered to represent the age distribution of the population of Japan.[14,15] In the present study, we analyzed data from 3,080 people enrolled in the Hisayama study in 2007.

The JDOPPS is part of the International Dialysis Outcomes and Practice Patterns Study, an international longitudinal study of hemodialysis patients. Patients in the JDOPPS were selected randomly from among representative dialysis facilities in Japan, and they appear to represent all hemodialysis patients in Japan. The design of the DOPPS is detailed elsewhere.[16] After we excluded data from hemodialysis patients whose dietary intake was not measured and those with a daily energy intake of less than 500 kcal or more than 4,000 kcal, data from 1,355 hemodialysis patients who participated in the third phase of the JDOPPS between 2005 and 2007 were available for analysis.

### The predictors

The methods regarding the predictors had four steps: (1) collection of data on food consumption, (2) identification of food groups, (3) computation of food-group scores, and (4) identification of dietary patterns. Those four steps are described in sequence below. We note that this method for identifying dietary patterns is based on foods and food groups, not on micronutrients, and that methods such as the one we used in this study are common in nutritional epidemiology.[17–20]

(1) Collection of data on food consumption (Hisayama study): Data on foods consumed were obtained using a brief self-administered diet-history questionnaire (the BDHQ).[21–23] The BDHQ is a 4-page structured questionnaire that contains questions about 58 foods and beverages, and allows the total energy intake and the intake of micronutrients to be estimated. Reports of previous studies indicate that food intake estimated using the BDHQ is consistent with intake measured using semi-weighted 16-day dietary records.[21,24] Food intake was measured with the BDHQ in the Hisayama study in 2007 and in the JDOPPS during the second year of JDOPPS enrollment, between 2006 and 2007.

(2) Identification of food groups (Hisayama study): To identify food groups, we conducted a principal components analysis (PCA). We used PCA with Promax rotation to reduce the results regarding the many foods listed in the BDHQ to a smaller set of food groups. That is, we used PCA to identify groups of foods that were eaten with approximately equal frequencies by the same people. We did those analyses with data from 3,080 participants in the Hisayama study. Here it is important to remember one similarity between PCA and other multivariate analyses: When the values of an independent variable are nearly the same among almost all participants, then that independent variable contributes little or no information to the results, and such variables should be deleted from the analyses. Therefore, in PCA it is common practice to delete items that vary by only small amounts between individuals [25], so for the PCA in this study we used 20 foods from the 58 in the BDHQ.

(3) Computation of food-group scores (Hisayama study and JDOPPS): After identifying food groups, we standardized the frequency of consumption of each food by using the mean and standard deviation in the Hisayama data. Then we used those standardized frequencies to compute food-group scores for each JDOPPS patient, and we used the residual method [26] to adjust those food-group scores for the total daily energy intake

(4) Identification of dietary patterns (JDOPPS): To identify dietary patterns in the JDOPPS patients, we used Ward’s method of cluster analysis[27] on the energy-adjusted food-group scores. Thus, the patterns we identified were based on the relative amounts of foods from each food group that the JDOPPS patients actually ate.

### The outcome

This study had one outcome, which was a composite of important adverse clinical events: hospitalization due to cardiovascular disease or death due to any cause. Cardiovascular disease included coronary heart disease, arrhythmia, congestive heart failure, cardiac valvular disease, cardiac myopathy, and pericarditis. The date and cause of hospitalization was ascertained approximately every 4 months in the JDOPPS.

### Analyses (associations between dietary patterns and the outcome)

Cox regression analysis was used to investigate relationships between dietary patterns and the composite outcome. Those relationships were expressed as hazard ratios. The time between the second year of food-intake measurement using the BDHQ and the composite outcome was studied first. Two models were used. In Model 1, the covariates considered in estimating the hazard ratio were age, sex, and hemodialysis duration. In Model 2, the covariates were body mass index, serum albumin, total daily energy intake, and comorbid conditions (coronary artery disease, congestive heart failure, cerebrovascular disease, peripheral vascular disease, and diabetes).

In a sensitivity analysis, we adjusted for hemoglobin level, the dose of erythropoietin-stimulating agent (ESA), and Kt/V, in addition to the covariates included in Model 2. In another sensitivity analysis, we adjusted for smoking habit in addition to the covariates included in Model 2.

All analyses were done with SAS 9.2 (SAS Institute, Cary, NC) and STATA 13.1 software (STATA, College Station, TX).

## Results

### Population characteristics

Table 1 shows the characteristics of participants in the Hisayama study and in the JDOPPS. We included 3,080 participants from the Hisayama study. The mean of their ages was 62.7 years, and 10.6% of them had diabetes. We also included 1,355 hemodialysis patients from the JDOPPS. The mean of their ages was 61.4 years, and 32.1% of them had diabetes. The mean duration of their dialysis was 7.6 years. The proportions of comorbidities, including diabetes and cardiovascular disease, were higher in the JDOPPS group than in the Hisayama group.

**Table 1:** Demographic and clinical characteristics of the participants in the Hisayama study and in the JDOPPS.

	**Hisayama(n = 3,080)**	**JDOPPS(n = 1,355)**
Mean (SD) age, years	62.7 (12.0)	61.4 (11.9)
Male (%)	43.6	61.4
Mean (SD) dialysis duration, years	NA	7.6 (7.2)
Mean (SD) BMI	23.1 (3.5)	21.1 (3.2)
Comorbid conditions (%)		
Diabetes	10.6	32.1
Coronary heart disease	6.0	41.3
Cerebrovascular disease	3.7	11.5
Other cardiovascular disease	8.2	30.6
Peripheral vascular disease	0.2	15.9
Cancer	7.5	9.4
Mean (SD) albumin, g/dL	4.2 (0.3)	3.8 (0.4)

JDOPPS: Japan Dialysis Outcomes and Practice Patterns Study, NA: not applicable, BMI: body mass index.

### Food groups (general–population results)

In the first PCA, “natto (fermented soybean)” had a moderate loading on 2 components. We therefore deleted “natto” and ran the PCA again. The first three components had eigenvalues greater than 1: 5.69, 1.53, and 1.35, which accounted for 28.4%, 7.8%, and 6.74% of the variance, respectively. As shown in Table 2, three food groups were identified. The first group included carrot & pumpkin, root vegetables, cabbage (cooked), mushrooms, seaweed, lettuce & cabbage (raw), potatoes, tofu (bean curd) & fried tofu, turnip (radish), and tomato. This we call the vegetables group. The second group included squid & octopus & shrimp & shellfish, dried fish, fatty fish, lean fish, and small fish with bones. This we call the fish group. The third group included ham, pork & beef, chicken, and eggs. This we call the meat group.

**Table 2:** Coefficients after Promax rotation (Principal Components Analysis, Hisayama data).

		**Component**		
**Food Item**		**1**	**2**	**3**
Carrot/pumpkin		0.798	−0.079	0.001
Root vegetables		0.754	−0.055	−0.022
Green leafy vegetables		0.705	−0.092	0.067
Cabbage (cooked)		0.686	−0.146	0.148
Mushrooms		0.636	0.058	0.013
Seaweed		0.591	0.135	−0.127
Lettuce/cabbage (raw)		0.543	−0.122	0.260
Potatoes		0.530	0.168	−0.057
Tofu (bean curd)/fried tofu		0.504	0.154	−0.026
Turnip (radish)		0.497	0.189	−0.106
Tomato		0.426	0.104	−0.003
Dried fish		−0.081	0.693	0.087
Fatty fish		0.027	0.630	0.116
Lean fish		0.103	0.594	−0.034
Small fish with bones		0.160	0.591	−0.179
Squid, octopus, shrimp, shellfish		−0.095	0.536	0.248
Ham		−0.135	0.042	0.718
Pork/beef		0.054	0.019	0.715
Chicken		0.057	0.121	0.564
Eggs		0.116	−0.012	0.474
Coefficents of correlation	1	1.000	0.430	0.267
	2		1.000	0.242
	3			1.000
Deleted food				
Natto				

Data on 20 foods were analyzed with principal components analysis (Promax rotation). The first three components had eigenvalues greater than 1: 5.69, 1.53, and 1.35, which accounted for 28.4%, 7.8%, and 6.74% of the variance.

### Dietary patterns in hemodialysis patients

Cluster analysis of the adjusted food-group scores revealed three clusters, which we call (1) “well-balanced diet”, (2) “unbalanced diet,” and (3) “other diet” (Table 3). Patients in the first of those three clusters, i.e. those whose diet was well-balanced, were those who ate approximately equal amounts of food from the meat, fish, and vegetable groups. Almost half of the JDOPPS patients had a well-balanced diet (49.2%). Patients in the second of the three clusters, i.e. those whose diet was unbalanced, were those who ate a much larger amount from the vegetable group than from the meat group and the fish group. They amounted to 14% of the JDOPPS patients.

**Table 3:** Adjusted food-group scores for each cluster (JDOPPS data).

		**Food-group score**		
**Cluster**	***n***	**Vegetables**	**Fish**	**Meat**
Well-balanced	666 49.2%	0.297 (0.460)	0.216 (0.936)	0.319 (0.874)
Unbalanced	189 14.0%	1.522 (0.454)	0.528 (0.809)	0.315 (0.838)
Other	500 36.9%	−0.971 (0.643)	−0.488 (0.945)	−0.544 (0.980)

Each food-group score was adjusted for total daily energy intake by the residual method [20]. Values in parentheses are standard deviations.

**Figure 1:**
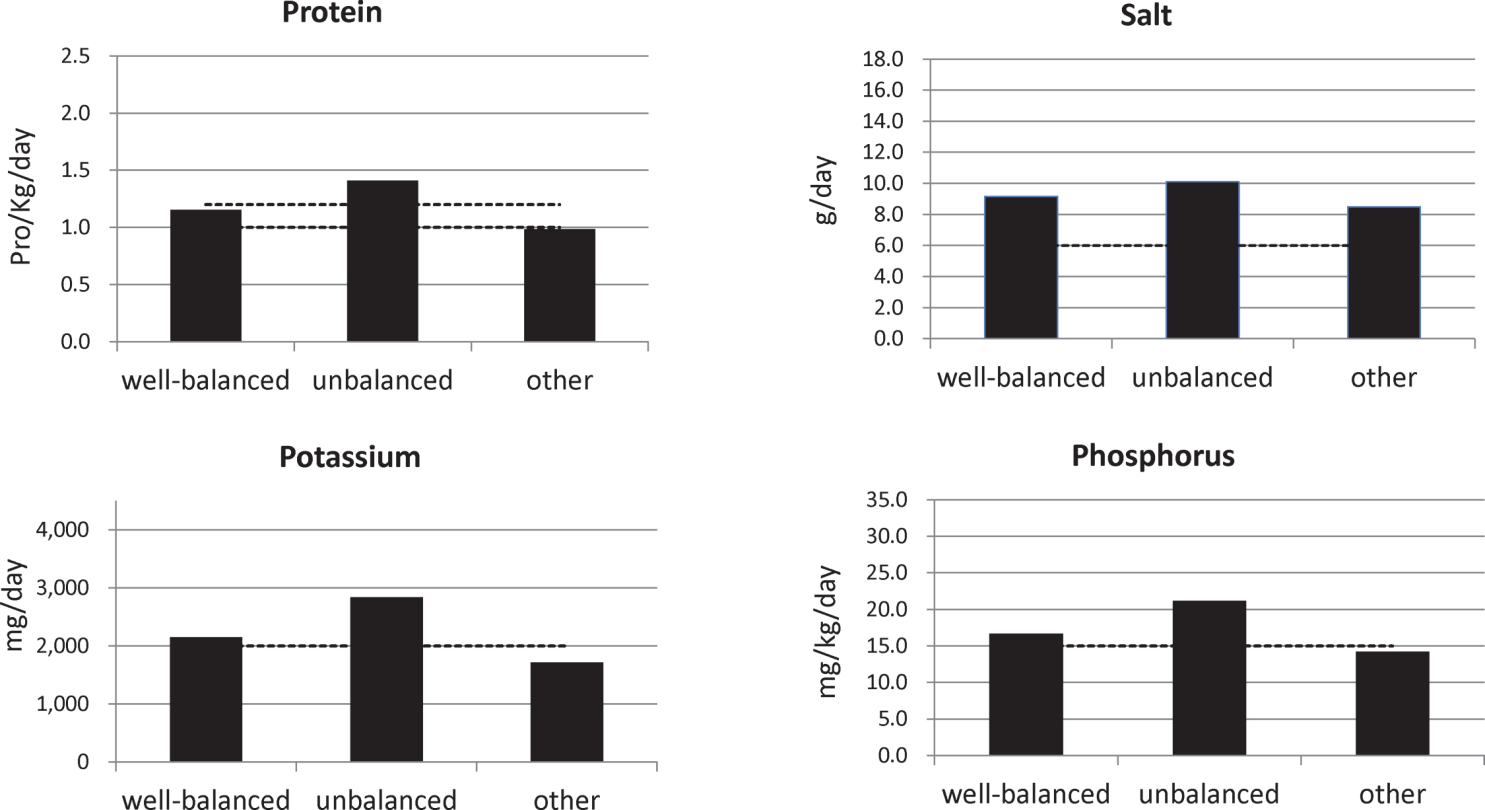
Micronutrient intake stratified by dietary pattern. Estimated micronutrient intake stratified by dietary pattern. Dotted lines show dietary standards according to Japan’s clinical guidelines (Dietary recommendations for chronic kidney disease, 2007, Japanese Society of Nephrology).

Fig. 1 shows the amounts of micronutrients for each cluster of JDOPPS patients. According to clinical guidelines, protein intake was within the prescribed range among those who ate a well-balanced diet, too high among those who ate an unbalanced diet, and too low among the others. [1] The mean salt intake was more than 6 g/day in all groups, and was highest among those who ate an unbalanced diet. Potassium intake was within the prescribed range among those who ate a well-balanced diet, too high among those who ate an unbalanced diet, and too low among the others. Phosphorus intake was similar to potassium intake.

### Patient characteristics by dietary pattern

Table 4 shows characteristics of the JDOPPS patients, stratified by the three dietary patterns. Patients who ate an unbalanced diet were older than those who ate a well-balanced diet, and fewer of them were male. Total daily energy intake, protein intake, salt intake, and potassium intake were highest among those whose diet was unbalanced.

**Table 4:** JDOPPS patient characteristics at baseline, by dietary pattern (n = 1,355).

	**Well-balanced (49.2%)**	**Unbalanced (14.0%)**	**Other (36.9%)**
Mean (SD) age, years	62.3 (11.8)	64.2 (11.9)	59.2 (11.5)
Male (%)	57.2	40.7	74.8
Mean (SD) dialysis duration, years	7.7 (7.4)	7.2 (7.2)	7.6 (7.0)
Mean (SD) BMI	21.2 (3.3)	20.2 (3.0)	21.5 (3.1)
Comorbid conditions (%)			
Diabetes	31.7	32.8	32.2
Coronary Heart Disease	44.3	41.8	37.2
Cerebrovascular Disease	12.8	12.7	9.4
Other Cardiovascular Disease	28.5	36.0	31.2
Peripheral Vascular Disease	15.6	22.2	14.0
Cancer	8.7	9.6	10.3
Mean (SD) serum albumin, g/dL	3.8 (0.4)	3.8 (0.5)	3.9 (0.4)
Mean (SD) phosphorus, mg/dL	5.6 (1.3)	5.3 (1.4)	5.6 (1.4)
Mean (SD) serum potassium, mEq/L	5.1 (0.8)	5.1 (0.8)	5.0 (0.8)
Mean (SD) energy intake, cal/Kg/day	1592 (563)	1707 (538)	1640 (656)
Mean (SD) protein intake, g/Kg/day	1.16 (0.51)	1.41 (0.59)	0.99 (0.49)
Mean (SD) salt intake, g/day	9.16 (3.32)	10.10 (3.21)	8.48 (3.60)
Mean (SD) potassium intake, g/day	2.15 (0.86)	2.84 (1.01)	1.72 (0.84)
Mean (SD) phosphorus intake, mg/day	883 (370)	1018 (376)	793 (395)

The “well-balanced diet” was characterized by approximately equal intake of the three food groups (fish, meat, and vegetables). The “unbalanced diet” was characterized by relatively large vegetable intake compared with meat and fish intake, and the “other diet” refers to other intake patterns.

### Association between dietary pattern and clinical outcomes in hemodialysis patients

Table 5 shows associations between dietary patterns and the composite outcome. In Model 1, which included adjustments for age, gender, and dialysis duration, the unbalanced diet was associated with a higher event rate than the well-balanced diet (adjusted hazard ratio [HR] 1.81, 95% CI 1.15–2.85). A similar association was seen in Model 2 (adjusted HR 1.90, 95% CI 1.19–3.04), that is, after adjustment for serum albumin, BMI, and total daily energy intake, in addition to the covariates included in Model 1.

**Table 5:** Dietary patterns and the composite outcome (JDOPPS data).

**Dietary patterns**	**Composite outcome rate (/100 person-years)**	**Model 1**	**Model 2**
		**Hazard ratio (95% CI)**	**Hazard ratio (95% CI)**
Well-balanced	7.4	Reference	
Unbalanced	10.3	1.81 (1.15–2.85)	1.90 (1.19–3.04)
Other	6.1	1.23 (0.82–1.83)	1.21 (0.81–1.82)

The composite outcome included hospitalization due to cardiovascular disease, and death due to any cause. Model 1: Adjusted for age, gender, and dialysis duration. Model 2: Adjusted for age, gender, dialysis duration, serum albumin, BMI, total daily energy intake, and comorbid conditions (diabetes, coronary heart disease, cerebrovascular disease, other cardiovascular disease, and peripheral vascular disease).

In the sensitivity analysis adjusted for the covariates included in Model 2 and also adjusted for hemoglobin level, ESA dose, and single-pool Kt/V, we also found a similar association between unbalanced diet and the composite outcome (adjusted HR 1.89, 95% CI 1.11–3.23). In the other sensitivity analysis, adjusted for the covariates included in Model 2 and also for smoking habit, we again found a similar association between unbalanced diet and adverse clinical events (adjusted HR 1.85, 95% CI 1.16–2.97).

## Discussion

Using PCA with data from a representative sample of the general population of Japan, we identified three food groups: meat, fish, and vegetables. We then found that hemodialysis patients could be said to have diets that were “well-balanced” or “unbalanced” with regard to those three food groups. (As noted previously, to identify dietary patterns based on foods or on food groups, as we did in this study, is common in nutritional epidemiology.[17–19]) The hemodialysis patients whose diet was unbalanced were more likely to have important clinical events. These findings suggest that limiting food portions, which is often recommended for hemodialysis patients to prevent severe adverse clinical outcome, is not enough. In addition to portion control, a diet that is balanced among meat, fish, and vegetables might help to prevent adverse outcomes.

Nutritional epidemiologic research in hemodialysis patients has largely focused on relationships between individual food items, micronutrients, and outcomes. For example, relationships between fish consumption, phosphate consumption, and outcomes in these patients have been reported.[7,28] However, hemodialysis patients do not eat only one specific food item, but rather various foods, and therefore dietary patterns should be determined on the basis of the combinations of foods that people actually eat. We began with PCA, from which we identified three groups of foods that are in fact eaten by people in Japan: meat, fish, and vegetables. We then used cluster analysis, from which we identified hemodialysis patients’ actual patterns of food consumption with reference to those groups. Those patterns (well-balanced, unbalanced, and other) were associated with important clinical outcomes.

In hemodialysis patients, adequate protein intake (1.0 to 1.2 g/kg per day), such as can be obtained from the meat and fish groups we identified, is recommended to counteract loss of protein via the dialysate.[29] Sufficient protein intake is critical to preventing malnutrition, but excessive protein intake may lead to hyperphosphatemia, which may in turn lead to cardiovascular events. Hemodialysis patients should also avoid excessive vegetable intake to prevent hyperkalemia, which, like hyperphosphatemia, is associated with cardiovascular events. It is therefore physiologically plausible that a diet well-balanced among food groups would be associated with good clinical outcomes, as was found in this study.

The present study had a number of strengths. First, the Hisayama study and the JDOPPS used representative samples of the general population of Japan and of hemodialysis patients in Japan, respectively. Therefore the findings should be generalizable to all hemodialysis patients in Japan. To the extent that differences in dietary patterns between hemodialysis patients in Japan and those in other countries can result in differences in clinical outcomes, the present findings might be used for nutritional research and possibly also for dietary recommendations to improve the prognosis of patients in, for example, the US and Europe. Second, the use of the BDHQ enabled us to measure food intake in clinical settings.[21–24] Third, results of the sensitivity analyses indicated that the association of dietary pattern with the composite outcome was robust with respect to hemoglobin level, ESA dose, Kt/V, and smoking habit.

One possible limitation of this study is that food intake was self-reported. Actual food intake might have differed from that estimated from the food-frequency questionnaire.[30] In particular, social-desirability bias might have caused hemodialysis patients, who were aware of their dietary proscriptions, to report inaccurately-low levels of food intake, and the estimated intake of micronutrients might therefore have been incorrect.

In summary, eating a diet that was not balanced among meat, fish, and vegetables was associated with important adverse clinical events, which suggests that hemodialysis patients should not only limit their food intake but should also strive for a proper balance among those three food groups.
